# Short-Term Changes in Tear Film Stability and Tear Volume Following the Application of Various DED Management Options in a Healthy Young Population

**DOI:** 10.3390/jpm15050173

**Published:** 2025-04-27

**Authors:** Belén Sabucedo-Villamarin, Jacobo Garcia-Queiruga, Laura Cacabelos-Torres, Maria J. Giraldez, Eva Yebra-Pimentel, Hugo Pena-Verdeal

**Affiliations:** 1 GI-2092-Optometry, Departamento de Fisica Aplicada (Area de Optometria), Universidade de Santiago de Compostela, Campus Vida s/n, 15701 Santiago de Compostela, Spainhugo.pena.verdeal@usc.es (H.P.-V.); 2 Instituto de Investigacion Sanitaria (IDIS), Travesia da Choupana S/N, 15701 Santiago de Compostela, Spain

**Keywords:** dry eye disease, dry eye management, artificial tears, eyelid cleansing wipes, ocular bath

## Abstract

**Background:** To determine short-term changes in tear film volume and stability after various treatments for dry eye disease in healthy participants. **Methods:** 36 healthy participants aged from 18 to 35 years were recruited for a single-session examination and randomly assigned to one of three treatment groups (1:1:1 treatment, 1:1 eye): Group 1 (artificial tears ‘Comfort Drops’), Group 2 (eyelid wipes ‘Systane Lid Wipes’), and Group 3 (ocular bath ‘Acuaiss’). Tear Meniscus Height (TMH) was assessed at baseline, 2, 5, 10, 15, and 20 min, and Non-Invasive Break-Up Time (NIBUT) at baseline, 2, 10, and 20 min, all using the OCULUS Keratograph 5M by one examiner. **Results:** Of the initially recruited participants, 35 were analyzed; one was excluded for reflex tearing. Group 1 (n = 12) showed a significant TMH increase at 2 min compared to 10 and 20 min (Friedman, *p* = 0.004; Bonferroni, *p* ≤ 0.028). Group 3 (n = 12) showed a significant increase at 2 and 5 min compared to baseline and decrease at 10 min against 2 and 5 min (Friedman, *p* < 0.001; Bonferroni, *p* ≤ 0.034). Group 2 (n = 11) showed no significant changes over time (Friedman, *p* = 0.108). NIBUT showed no significant differences at any time-point in any group (Friedman, *p* ≥ 0.231). Basal TMH differed between groups (ANOVA, *p* = 0.048), but post hoc analysis found no significance (Bonferroni, all *p* ≥ 0.088). No significant differences in TMH at other time-points (Kruskal–Wallis/ANOVA, *p* ≥ 0.265) or in NIBUT between groups (Kruskal–Wallis/ANOVA, *p* = 0.108) were found. **Conclusions**: In healthy participants, artificial tears and ocular baths temporarily increase TMH, while eyelid wipes do not. Neither has an immediate impact on NIBUT.

## 1. Introduction

Dry Eye Disease (DED) is a complex and multifactorial condition characterized by a disruption of tear film homeostasis, which is essential for maintaining a functional ocular surface [1]. This disease is associated with a wide range of ocular symptoms, and is caused by several key factors, including tear hyperosmolarity, ocular surface inflammation and damage, and abnormal neurosensory function [1,2,3,4]. The symptoms experienced by individuals with DED are varied but commonly include persistent ocular discomfort, such as pain, burning, stinging, and grit or foreign body sensation in the eyes [5,6]. Additionally, many patients report a noticeable decline in visual acuity, often describing their vision as poor or blurred [7]. This visual disturbance is directly linked to the compromised tear film, which disrupts the smooth optical surface of the eye, causing fluctuations in vision quality. This condition has a high prevalence, as evidenced by recent studies which report an overall rate of 11.59%. However, in certain populations, such as the elderly, postmenopausal women, or a combination of both, the prevalence can be as high as 50% [2,4,8]. The high prevalence of DED in today’s society underlines the need to deepen the knowledge and personalized treatment of this condition due to its nature as an important public health problem.

The ocular complaints due to DED significantly impact on the quality of life of those affected, often interfering with daily activities [8,9]. In addition to its clinical implications, DED represents a substantial economic burden for individuals and healthcare systems [10,11,12]. Recent studies estimate the annual cost to patients at USD 375, encompassing expenses such as over-the-counter products, prescription medications, and medical consultations [13]. On a larger scale, the economic impact on healthcare systems is profound, with an estimated annual cost of USD 3.84 billion, driven by increased healthcare utilization and indirect costs, such as lost productivity [13]. This dual burden highlights the need for effective and accessible treatments to alleviate both the physical and financial costs of the disease.

A wide range of treatment options are available to manage and alleviate the symptoms of DED, ranging from mild interventions to more aggressive approaches. Initial options include omega-3 supplements, artificial tears or advanced therapies, such as autologous serum drops [14,15,16,17]. In the most severe cases, or cases resistant to conventional management therapies, immunomodulatory and anti-inflammatory medications such as corticosteroids and cyclosporine may be used, and surgery may be required, including permanent occlusion of the lacrimal punctum to reduce tear drainage and maintain ocular surface hydration [18,19,20,21]. Despite the availability of various treatment options, artificial tears remain the most commonly used therapy and have been the focus of extensive research [14,22]. Artificial tears, comprising components such as hyaluronic acid, liposomes, or hypromellose, have been demonstrated to enhance the stability, viscosity, and lubricity of the tear film [14,15,16,22]. These properties effectively reduce ocular discomfort and improve visual quality for many patients. However, despite their popularity, artificial tears address only part of the range of the therapeutic options [14,15,23]. Other options for DED management, such as eyelid hygiene using cleansing lid wipes or ocular baths, remain understudied regarding the changes they can induce in the tear film and ocular surface [17,24,25,26]. An important gap in current knowledge is the lack of robust clinical trials evaluating the effects of these treatments on the tear film. Therefore, the aim of the present study was to investigate changes in tear film volume and stability over a short period of time following the use of artificial tears, eyelid cleansing wipes, and eye baths in a sample of healthy participants. Understanding how these different DED treatment options affect the tear film in healthy subjects would help clinicians to better recommend one treatment or another to restore the ocular homeostasis in DED suffers. To this end, the study seeks to lay the foundation for improving both current and future treatments for DED, by personalizing their dosage and methods of administration based on the specific parameters of each patient, ultimately enhancing their long-term prognosis.

## 2. Materials and Methods

### 2.1. Sample and Study Design

Prior to the recruitment process, the sample size of each group was calculated based on the Tear Film and Ocular Surface Society proposal in the Dry Eye Workshop II Diagnostic Methodology report by using the software PS Power and Sample Size Calculations Version 3.1.2 (Copyright© by William D. Dupont and Walton D. Plummer) [4,27]. The standard deviations (SDs) reported in the literature on Tear Meniscus Height (TMH) and Non-Invasive Break-Up Time (NIBUT) were assumed to be 0.05 mm and 3 s, respectively. To have 80% power for a significance level of α = 0.05 (Type I error associated) the minimum number of participants required in each group was 6 and 10, respectively. The highest of those values was selected as the reference for determining the minimum sample size (10 participants) to ensure a more robust study.

Based on the sample size calculation, a total of 36 healthy participants aged between 18 and 35 years old were initially recruited from patients attending the optometry service of the institution for a routine eye examination who met the following inclusion criteria. No one had a prior history of ocular diseases (other than dry eye complaints), ocular surgery, systemic or autoimmune diseases, was pregnant or breast-feeding, wore contact lenses or was under medical treatment. All participants provided their written consent, and the study protocol was approved by the Bioethics Committee of the institution (Code: USC-08/2021) and adhered to the tenets of the Declaration of Helsinki.

The total sample was randomly distributed among the three different groups according to DED treatment with a 1:1:1 allocation ratio for treatment group assignment and a 1:1 allocation ratio per eye according to the order of admission: artificial tears ‘Comfort Drops’ (CooperVision Inc., Pleasanton CA, USA) were assigned to Group 1, eyelid cleansing wipes ‘Systane Lid Wipes’ (Alcon Inc., Fort Worth, TX, USA) were assigned to Group 2, and ocular bath ‘Acuaiss’ (Disop, Madrid, Spain) was assigned to Group 3.

Following the assignment of treatment and distribution to each group, the study protocol was conducted in a single session with a baseline measurement and measurements at different time-points after application of the DED treatment. All measurements in the study were conducted between 9:00 AM and 2:00 PM by a single examiner to reduce variability associated with circadian rhythms and interobserver differences [28,29,30]. At baseline, TMH and NIBUT were both measured [31]. Following the administration of the DED treatment, all procedures were always performed in the same order, first TMH and then NIBUT [27]. TMH was measured at 2, 5, 10, 15, and 20 min and NIBUT was measured at 2, 10, and 20 min. The entire study protocol was conducted under controlled environmental conditions of light, temperature (20–23 °C), and humidity (50–60%).

### 2.2. DED Management Treatments and Application Instructions

#### 2.2.1. Artificial Tears

The artificial tear used was CooperVision Comfort Drops, in 20 mL multidose format, which is composed of polyhexadine 0.0001%, hydroxypropyl methylcellulose (HPMC), ethylenediaminetetraacetic acid (EDTA), sodium phosphate, poloxamer, sodium chloride and water. Three drops were instilled in the studied eye of each participant of Group 1.

#### 2.2.2. Eyelid Cleansing Wipes

The eyelid cleaning wipes used were Systane Lid Wipes, which are composed of hydrogenated polietilenglicol (PEG)-200 glyceryl palmate, disodium laureth sulfosuccinate, cocamidopropylamine oxide, PEG-80 glyceryl cocoate, benzyl alcohol, and disodium EDTA and water. The manufacturer’s instructions for using the eyelid cleansing wipes were followed. The manufacturer suggests rubbing the closed sachet containing the wipe to create foam. The wipe was then applied twice along the free edge of the lower eyelid of the studied eye of each participant of Group 2.

#### 2.2.3. Ocular Bath

The ocular bath used was Acuaiss, which is composed of sodium chloride, disodium phosphate, monosodium phosphate, hyaluronic acid, and 0.0002% poly-hexamethylene biguanide in purified water. The manufacturer’s instructions for using the ocular bath were followed. The product included a container that should be filled with the ocular bath. Each participant in Group 3 kept the studied eye open and in contact with the ocular bath inside the designated container for 30 s, during which blinking and eye movement were allowed.

### 2.3. Evaluation Procedures

#### 2.3.1. Tear Film Volume Assessment

The tear film volume was assessed by measuring the TMH using the Keratograph 5M (Oculus Optikgerate GmbH, Wetzlar, Germany) [31,32,33]. Participants were positioned correctly and instructed to look straight ahead and blink normally. The device is equipped with a specific function that uses white-light illumination to visualize the tear meniscus. Following this, three images of the tear meniscus were captured. To ensure precise measurements, the examiner was required to position the upper and lower limits in the middle third, aligning them with the pupil. The device then provides the measurement of the distance between these limits in millimeters, which corresponds to the TMH.

#### 2.3.2. Tear Film Stability Assessment

Tear film stability was assessed by measuring the NIBUT using the Keratograph 5M, which uses the placid disc principle [31,34,35]. Immediately following the TMH measurement, the participants were instructed to maintain the same position. Prior to each measurement, participants were instructed to look at a red dot, blink twice and keep their eyes open for as long as possible. At this point, the device begins recording and automatically calculates the time it takes for the first tear film break to occur.

### 2.4. Statistical Analysis

SPSS statistical software v.25.0 for Windows (SPSS Inc., Chicago, IL, USA) was used for the data analyses. Statistical significance was set at *p* < 0.05 for all the analyses. First, the normal distribution of the data for each group was checked using the Shapiro–Wilk test for samples at each time-point. TMH showed a normal distribution at every time-point for all groups (all *p* ≥ 0.089); however, TMH at 2 min for group 2, TMH at 10 min for groups 1 and 2, and TMH at 15 min for groups 1 and 2 showed a non-normal distribution (all *p* ≤ 0.045). NIBUT showed a normal distribution at every time-point for all groups (all *p* ≥ 0.085); however, NIBUT at basal for group 2, NIBUT at 2 min for group 1, and NIBUT at 10 min for group 3 showed a non-normal distribution (all *p* ≤ 0.039) [36,37].

The Friedman test was conducted to assess differences within each time-point in every group [37]. Also, Bonferroni correction was applied to adjust for multiple comparisons. Finally, on one hand, independent-sample ANOVA was conducted to compare groups at each time-point that were normally distributed, and Bonferroni post hoc tests were performed for paired analysis between groups [38,39]; on the other hand, the independent-sample Kruskal–Wallis test was conducted to compare groups at each time-point that were non-normally distributed, and Bonferroni correction for multiple comparisons was also applied.

## 3. Results

Even though the total sample recruited was 36 participants, only 35 were included in the final analysis, as one participant was excluded due to reflex tearing that interfered with the measurements. The demographic characteristics of the studied population, age and sex, and the descriptive statistics for each group are shown in Table 1.

The Chi-square (*p* = 0.871) and Kruskal–Wallis (*p* = 0.659) tests showed no significant differences in sex and age distribution among the three groups, confirming the sample’s homogeneity.

### 3.1. Intra-Group Analysis of TMH Across Different Time-Points

**Figure 1:**
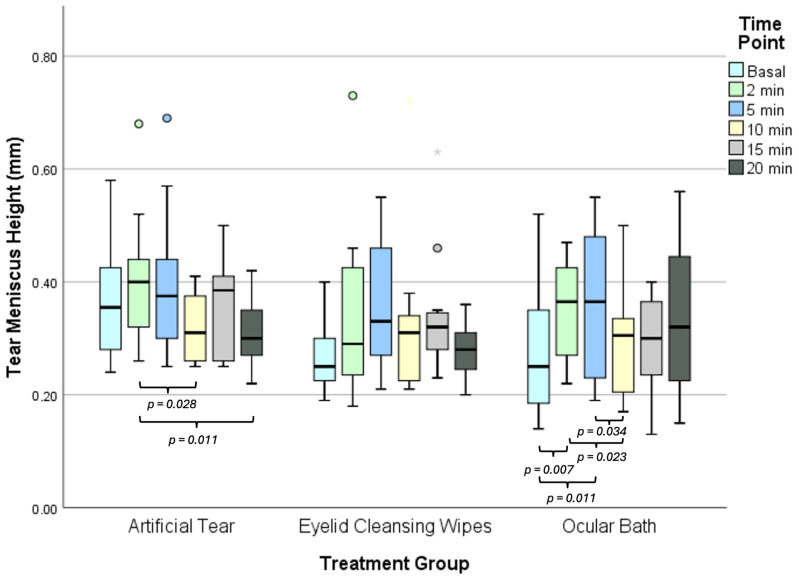
Boxplot graph represents TMH values across different time-points for each DED treatment group. The box illustrates the sample’s interquartile range (25th–75th percentile), with the black line representing the mean. Dots indicate outliers, exceeding 1.5 box lengths beyond the quartiles, while asterisks denote extreme outliers, surpassing 3 box lengths. DED: Dry Eye Disease; TMH: Tear Meniscus Height.

Statistically significant differences in the TMH values at different time-points were observed in Groups 1 and 3 (Friedman test, both *p* ≤ 0.004) but not in Group 2 (Friedman test, *p* = 0.108). In Group 1, pairwise analysis showed a statistically significant decrease in TMH values at 2 min after tear instillation compared to 10 min (Bonferroni post hoc, *p* = 0.028; Figure 1) and 20 min (Bonferroni post hoc, *p* = 0.011; Figure 1). In Group 2, no pairwise differences between time-points were found (Bonferroni post hoc, all *p* ≥ 0.292). In Group 3, pairwise analysis revealed significant differences between the baseline and TMH values at 2 min and 5 min post-application of the ocular bath (Bonferroni post hoc, both *p* ≤ 0.011; Figure 1), as well as between 10 min and 2 min and 5 min post-application (Bonferroni post hoc, all *p* ≤ 0.034; Figure 1).

### 3.2. Intra-Group Analysis of NIBUT Across Different Time-Points

**Figure 2:**
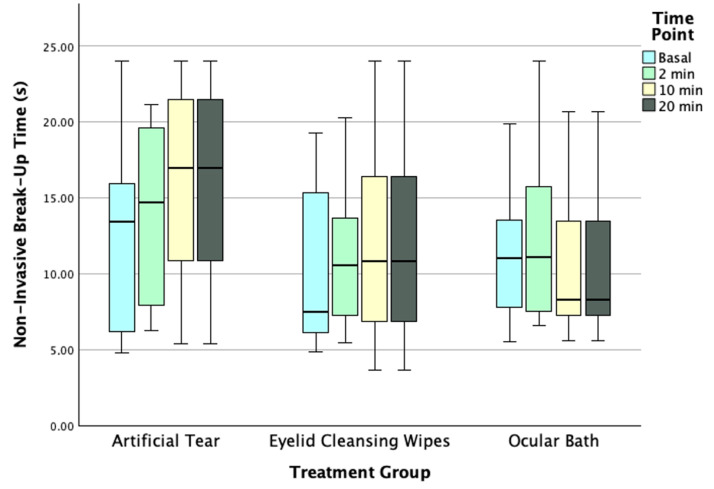
Boxplot graph represents NIBUT values across different time-points for each DED treatment group. The box illustrates the sample’s interquartile range (25th–75th percentile), with the black line representing the mean. DED: Dry Eye Disease; NIBUT: Non-Invasive Break-Up Time.

No statistically significant differences were found in the NIBUT values across the different time-points in all three groups (Friedman test, all *p* ≥ 0.231). Also, pairwise analysis between time-points showed no differences in Group 1 (Bonferroni post hoc, all *p* ≥ 0.239), Group 2 (Bonferroni post hoc, all *p* = 1.000), and Group 3 (Bonferroni post hoc, all *p* = 1.000) (Figure 2).

### 3.3. Inter-Group Analysis of TMH at Different Time-Points

Statistically significant differences in basal TMH values were observed between treatment groups (ANOVA, *p* = 0.048). However, pairwise analysis between treatment groups showed no statistically significant differences (Bonferroni post hoc, all *p* ≥ 0.088). No statistically significant differences in TMH values for any other time-point between treatment groups were found (Kruskal–Wallis or ANOVA, all *p* ≥ 0.265).

### 3.4. Inter-Group Analysis of NIBUT at Different Time-Points

No statistically significant differences were found in NIBUT between groups across the different time-points post-application, regardless of the treatment used (Kruskal–Wallis or ANOVA, all *p* ≥ 0.108). Also, pairwise analysis between treatment groups showed no statistically significant differences in any of the time-points (Bonferroni post hoc, all *p* ≥ 0.130).

## 4. Discussion

Due to the multifactorial nature of DED and its high prevalence, numerous treatments and therapies have been developed for its management [15,16,40]. These aim to alleviate a wide range of symptoms, from burning and itching to blurred vision. However, the vast array of available options, combined with their high cost, often makes it difficult for both patients and clinicians to determine the most suitable treatment. Given the potential socio-economic impact, it is crucial to establish clear treatment criteria, which can be informed by studying the changes these therapies induce in the tear film and ocular surface. Therefore, the present study was conducted in a healthy young population and assessed short-term changes in tear film volume and stability following the use of three commercially available therapeutic approaches for DED: artificial tears, eyelid cleansing wipes, and ocular baths. A comparative evaluation was conducted in a healthy young population, examining the effects of each treatment on tear quantity and quality. The findings aim to provide evidence of efficacy of these interventions in restoring ocular homeostasis in healthy subjects, thereby supporting clinicians in selecting the most appropriate and personalized management strategies tailored to the various types of DED.

The present study showed that the instillation of artificial tears varies the tear volume estimated by measuring TMH over a short period of time. The current results indicate that TMH decreases at 10 min, increases slightly at 15 min, and then decreases again at 20 min after artificial tear instillation. After the administration of these HPMC-based artificial tears, the tear volume increases slightly initially but then returns rapidly to baseline. The increase in TMH values may be due to the volume of the instilled drop itself, and an excessive tear volume in the ocular surface leads to increased tear drainage. Nevertheless, the artificial tears composition also plays an important role, as reported by Sánchez-González et al. [23] who studied two types of artificial tears, a Liposome Crosslinked Hyaluronic Acid (LCHA) artificial tear and a standard hyaluronic acid artificial tear. In their study, an increment in TMH values after 30 and 45 min since the instillation of LCHA artificial tears was found. However, they did not find differences in TMH at any time-point in a group of participants that used standard hyaluronic acid artificial tears. These findings support the hypothesis that artificial tear composition plays a role in tear film volume changes and tear drainage.

Furthermore, regarding the stability of the tear film, the present study found no statistically significant differences. The current findings suggest that tear film remains unchanged after artificial tear instillation. This outcome may be attributed to the HPMC-based artificial tear used in the present study compared to other investigations, where artificial tears with diquafosol sodium, hyaluronic acid or liposomes showed an increment in tear film stability values in short periods of time [23,41,42]. In the report of Sánchez-Gonzalez et al. [23], they found a significant increase in NIBUT values after 30 and 45 min compared to baseline in the LCHA group. However, in the standard hyaluronic acid artificial tear group they did not find differences between the basal measurement and 30 min after instillation, but it increased 45 min after instillation. Once again, these findings highlight the importance of the components based on each artificial tear. On the other hand, a study by Lievens et al. [43] found an improvement in tear film stability (measured by fluorescein break-up time) over a 30-day period with two different artificial tears based on another cellulose-derived compound (both based on carboxymethylcellulose). As stated, the current report did not find differences in NIBUT values in a short period of time with an HPMC-based artificial tear. Gagliano et al. [31] also studied an HPMC-based artificial tear in a DED population and found a decrease in tear film stability after 21 days of using it, suggesting that HPMC-based artificial tears could not benefit ocular health and tear film stability. This could be explained by a previous study where cellulose-based artificial tears containing chelating agents, such as EDTA, as the HPMC-based artificial tear used in the present study, have high osmolality values [44]. Therefore, the instillation of these artificial tears also produces an increase in the osmolarity in the tear film approaching hyperosmolarity. This triggers a compensatory cascade on the ocular surface to counteract the increase, initially leading to a transient rise in tear production and, consequently, an increase in tear drainage. In the end, these compensatory events ultimately disrupt the balance of the tear film, leading to its destabilization [3].

For the eyelid cleansing wipes in the present study, no significant differences were found in either tear volume or tear stability after application at any time-point. The use of these or similar products as eyelid cleansers has been described in various studies as important in the management of patients with blepharitis, relieving symptoms by removing debris from the lashes and in some cases even unblocking the meibomian glands [17,24,25,45]. However, there is a limited number of studies describing the changes that can occur in the tear or ocular surface. Only the study of Ngo et al. [26] assessed changes in both TMH and NIBUT, which found that TMH remained unchanged, while NIBUT increased after 1 and 3 months of continued treatment. Importantly, although this is the only study to date evaluating these ocular parameters, they combined the use of lid margin hygiene, artificial tears, and nutritional supplements. This highlights the need for further research to explore the effects of lid margin hygiene, when used as a single treatment, on ocular surface parameters both immediately after application and over a period of sustained use. Proper lid margin hygiene is known to be crucial in preventing conditions like blepharitis and meibomian gland dysfunction, both of which are closely associated with evaporative DED [46]. A deeper understanding of its impact would enable clinicians to more accurately evaluate the role of lid hygiene in DED management and refine treatment strategies accordingly.

In the case of the use of ocular baths, a similar situation applies, as to the authors’ knowledge, no previous studies have assessed their effects on symptoms or signs, either in the short or long term. This reinforces the findings of the present study, which showed no statistically significant differences in NIBUT at any measurement point. However, significant differences were observed in TMH between the baseline measurement and those at 2 and 5 min, as well as between the 2 and 10 min. These results suggest that after the application of the ocular bath, tear volume increases rapidly before returning to baseline, while tear stability remains stable.

The clinical significance of these findings lies in the observation that a short-term increase in TMH has positive implications for patients with aqueous-deficient dry eye, as it contributes to restoring tear volume to normal levels, suggesting that treatments such as artificial tears or eye baths may be beneficial for this patient group. In contrast, NIBUT remained unchanged throughout the observation period, regardless of the treatment applied, indicating that tear film stability does not improve in the short term. Consequently, these treatments may not be appropriate for patients with evaporative dry eye, in whom tear film stability is significantly compromised. However, to support this with greater confidence, long-term studies are needed to assess this over time. Taken together, these findings underscore the importance of considering the specific subtype of DED, when selecting treatment approaches, and they support the potential for a more personalized, subtype-specific management of the condition.

One of the greatest strengths of this study is the evaluation of tear quality parameters following the use of eyelid cleansing wipes and ocular baths, given the lack of similar studies in the existing literature. The present study provides valuable insights into how these treatments affect tear film stability and volume, contributing to a better understanding of their potential role in ocular surface health. Additionally, these results provide insight into how these tear parameters may be affected in the first few minutes after instillation, as most studies assess changes over days or months. However, a key limitation is that this study was conducted on a young population, which may not fully represent the effects that these treatments could have on individuals with DED and even in other age groups. Furthermore, although each group has been estimated according to calculation of the sample size, the overall sample size may be considered small. This may limit the generalizability of the results. Future research should focus on assessing these parameters in patients with DED and even in other age groups to determine whether the observed effects are consistent across different populations. More studies could help refine treatment recommendations and better tailor management strategies for affected individuals. In addition, improving treatment recommendations could have significant economic benefits, as more targeted approaches could help reduce unnecessary spending on less effective treatments, leading to better resource allocation and overall cost savings.

## 5. Conclusions

In conclusion, the present study showed that no short-term changes in tear film stability were observed after the use of different commercially available treatments for DED in a healthy population. However, there was a short-term increase in tear volume following the use of eye baths and, to a lesser extent, HPMC-based artificial tears.

## Figures and Tables

**Table 1:** Demographics and descriptive statistics of the variables of each group. IQR: Interquartile Range. Min: Minutes. SD: Standard Deviation. NIBUT: Non-Invasive Break-Up Time. TMH: Tear Meniscus Height.

			Group 1 (Artificial Tears)	Group 2 (Eyelid Cleansing Wipes)	Group 3 (Ocular Bath)
n			12	11	12
Age (Mean ± SD)			22.33 ± 2.15	22.55 ± 1.21	23.08 ± 3.12
Sex (% women)			66.67%	72.73%	75.00%
TMH	Basal	Mean ± SD	0.364 ± 0.103	0.270 ± 0.071	0.277 ± 0.114
		Median (IQR)	0.355 (0.16)	0.247 (0.09)	0.253 (0.18)
	2 min	Mean ± SD	0.403 ± 0.116	0.343 ± 0.160	0.353 ± 0.087
		Median (IQR)	0.397 (0.13)	0.290 (0.22)	0.367 (0.16)
	5 min	Mean ± SD	0.394 ± 0.130	0.365 ± 0.125	0.360 ± 0.130
		Median (IQR)	0.377 (0.17)	0.327 (0.29)	0.368 (0.25)
	10 min	Mean ± SD	0.317 ± 0.061	0.326 ± 0.145	0.291 ± 0.097
		Median (IQR)	0.310 (0.12)	0.313 (0.13)	0.307 (0.13)
	15 min	Mean ± SD	0.351 ± 0.088	0.344 ± 0.113	0.298 ± 0.085
		Median (IQR)	0.387 (0.16)	0.320 (0.09)	0.297 (0.15)
	20 min	Mean ± SD	0.310 ± 0.062	0.281 ± 0.052	0.345 ± 0.138
		Median (IQR)	0.300 (0.10)	0.283 (0.09)	0.318 (0.24)
NIBUT	Basal	Mean ± SD	12.580 ± 6.158	10.351 ± 5.548	10.979 ± 4.125
		Median (IQR)	13.432 (10.37)	7.457 (11.28)	11.047 (6.62)
	2 min	Mean ± SD	13.997 ± 5.951	11.156 ± 4.751	12.180 ± 5.381
		Median (IQR)	14.657 (11.84)	10.577 (8.24)	11.107 (9.03)
	10 min	Mean ± SD	13.397 ± 5.798	11.581 ± 6.920	11.010 ± 5.025
		Median (IQR)	13.797 (8.86)	9.983 (10.45)	10.068 (5.13)
	20 min	Mean ± SD	15.944 ± 6.535	12.040 ± 6.759	10.725 ± 4.805
		Median (IQR)	16.967 (11.15)	10.833 (10.96)	8.315 (6.69)

## Data Availability

The original contributions presented in this study are included in the article. Further inquiries can be directed to the corresponding authors.
